# Editorial Comment to Venous reconstruction using a Y‐shaped saphenous vein in kidney transplantation: A report of three cases

**DOI:** 10.1002/iju5.12285

**Published:** 2021-03-23

**Authors:** Tomokazu Shimizu

**Affiliations:** ^1^ Department of Transplant Surgery Kidney Center Toda Chuo General Hospital Saitama Japan; ^2^ Department of Urology Kidney Center Toda Chuo General Hospital Saitama Japan; ^3^ Department of Urology Tokyo Women’s Medical University Tokyo Japan

In this issue of the *IJU* *Case Reports*, Miyauchi *et al*. reported three living‐donor kidney transplant cases which the two donor right renal veins were reconstructed using a major Y‐shaped saphenous vein.^1^

In the case of living‐donor renal transplantation, the donor left kidney is usually selected because the right kidney has shorter venous length than the left kidney.^2^ If there are minor abnormalities in the donor right kidney, the normal donor left kidney is preserved. In these cases, the donor right kidney is tended to be used.

The shorter length of the allograft right renal vein is relevant to greater technical difficulty at a point of the venovenostomy.^2^, ^3^ When the allograft renal vein is short, one method is ample mobilization of the recipient’s common and external iliac veins and/or lateralization of the external iliac vein to the external iliac artery. Another method is the use of vein grafts to extend the length of the allograft renal vein.^3^

To lengthening the allograft short renal vein, some techniques have been performed, including a deceased donor’s vena cava patch, a donor’s or recipient’s gonadal vein, a deceased donor’s iliac vein, a superficial femoral vein graft, and a saphenous vein graft.^2^, ^3^, ^4^ A saphenous vein graft is a good material, but the difference in the diameter of this vein as compared with the renal vein causes technical difficulties.^5^ We previously mentioned renal vein lengthening using the saphenous vein in a case of short right allograft renal vein.^4^ Spiral anastomosis was performed with the longitudinally cut saphenous vein fragment to form a tubular shape, enabling anastomosis with the right allograft renal vein to achieve lengthening.^4^

When there are two veins, as reported in Miyauchi *et al*.’s study, this Y‐shaped saphenous vein graft seems to be effective in preventing outflow obstruction and to achieve the lengthening of the right allograft renal vein.^1^

I would like to use the Y‐shaped saphenous vein graft in case of short and multiple allograft renal veins.

## Conflict of interest

The author declares no conflict of interest.
